# Obesity alters adipose tissue response to fasting and refeeding in women: A study on lipolytic and endocrine dynamics and acute insulin resistance

**DOI:** 10.1016/j.heliyon.2024.e37875

**Published:** 2024-09-14

**Authors:** Lenka Rossmeislová, Eva Krauzová, Michal Koc, Marek Wilhelm, Viktor Šebo, Zuzana Varaliová, Veronika Šrámková, Moniek Schouten, Petr Šedivý, Petr Tůma, Jan Kovář, Dominique Langin, Jan Gojda, Michaela Šiklová

**Affiliations:** a Department of Pathophysiology, Centre for Research on Nutrition, Metabolism and Diabetes, Third Faculty of Medicine, Charles University, Prague, Czech Republic; b Franco-Czech Laboratory for Clinical Research on Obesity, Third Faculty of Medicine, Charles University, Prague and Université Toulouse III - Paul Sabatier (UPS), Toulouse, France; c Department of Internal Medicine, Third Faculty of Medicine, Charles University and Královské Vinohrady University Hospital, Prague, Czech Republic; d Department of Movement Sciences, Exercise Physiology Research Group, KU Leuven, Leuven, Belgium; e Institute for Clinical and Experimental Medicine, Prague, Czech Republic; f Department of Hygiene, Third Faculty of Medicine, Charles University, Prague, Czech Republic; g Institute of Metabolic and Cardiovascular Diseases, I2MC, University of Toulouse, Inserm, Toulouse III University - Paul Sabatier (UPS), Toulouse, France; h Centre Hospitalier Universitaire de Toulouse, Toulouse, France; i Institute Universitaire de France (IUF), Paris, France

## Abstract

Fasting induces significant shifts in substrate utilization with signs of acute insulin resistance (IR), while obesity is associated with chronic IR. Nonetheless, both states substantially influence adipose tissue (AT) function. Therefore, in this interventional study (NCT04260542), we investigated if excessive adiposity in premenopausal women alters insulin sensitivity and AT metabolic and endocrine activity in response to a 60-h fast and a subsequent 48-h refeeding period. Using physiological methods, lipidomics, and AT explants, we showed that obesity partially modified AT endocrine activity and blunted the dynamics of AT insulin resistance in response to the fasting/refeeding challenge compared to that observed in lean women. AT adapted to its own excess by reducing lipolytic activity/free fatty acids (FFA) flux per mass. This adaptation persisted even after a 60-h fast, resulting in lower ketosis in women with obesity. This could be a protective mechanism that limits the lipotoxic effects of FFA; however, it may ultimately impede desirable weight loss induced by caloric restriction in women with obesity.

## 1 Introduction

Adipose tissue (AT), the largest lipid reservoir in the body, is the key energy source during starvation or physical activity. The ability to store large amounts of energy in AT is a distinct advantage that allows people to survive extended periods with low caloric intake. Therefore, a phenotype characterized by increased adiposity during periods of food excess has been a desirable sign of well-being for most of human history. Today, however, with periods of food restriction virtually absent in the Western world, AT accumulation leading to obesity poses a major health threat by impairing cardiometabolic functions. In particular, obesity is an important risk factor for the development of chronic insulin resistance (IR), which precedes type 2 diabetes [1,2].

It is reasonably well accepted that the health risks of obesity are attributable to the metabolic dysfunction of AT. Many studies comparing lean and obese subjects or analyzing the effect of weight loss in the obese have reported that people with obesity have higher basal lipolytic activity (reviewed in Ref. [3]), resulting in higher levels of circulating free fatty acids (FFA), which damage non-adipose cells (the phenomenon is referred to as lipotoxicity) and trigger insulin resistance [2,4]. This widely accepted concept was challenged in 2011 by Karpe et al. In their review of the relationship between FFA levels, IR, and obesity, they found that circulating FFA levels were not clearly correlated with AT mass [5]. Also, more than 30 years of systematic research by S. Klein's group has repeatedly shown that the lipolytic activity of AT decreases with increasing adiposity [[6], [7], [8]]. Nonetheless, obesity appears to be linked to IR development via several other mechanisms, including immune and endocrine AT activity and exosome production [1]. Additionally, obesity may also compromise AT anabolic processes, e.g., FFA re-esterification and lipogenesis [9]. Still, about 7 % of the obese remain metabolically healthy (although this state is not always permanent) with preserved insulin sensitivity [10]. Thus, although it is clear that AT adapts its metabolic function to various physiological stimuli, it also adapts with respect to its own mass. The interactions between and among these adaptations, as well as their consequences, have are yet to be fully elucidated.

One of the highly physiological models that could provide further information on the metabolic and endocrine activity of AT and its relationship with IR is the model of prolonged fasting followed by refeeding, which fundamentally affects AT metabolism, overall insulin sensitivity, and substrate partitioning [11,12]. Currently, there is a lack of studies analyzing the impact of fasting in combination with refeeding, especially on women with differing adiposity. Our study used premenopausal women to investigate the impact of adiposity on the metabolic adaptations to a 60-h fast and subsequent 48-h refeeding, with a particular focus on measures of AT metabolism and insulin sensitivity.

## 2 Results

### 2.1 Measures of adiposity and lipid profiles at baseline and in response to the intervention

**Supplemental Figure S1:**
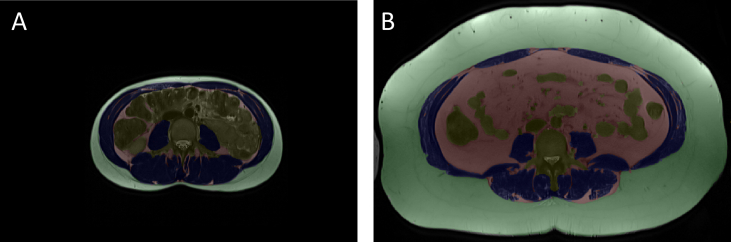
Supplemental Figure S1. MRI images used for analysis of truncal fat depots, related to Table 1 and Supplemental Table 1. Representative MRI scans with segmentation masks (green - subcutaneous adipose tissue, blue - muscle, red - visceral adipose tissue, yellow - bones and abdominal organs) used to evaluate subcutaneous and visceral adipose tissue deposition. A. lean woman, B. woman with obesity. The scale is the same for both subjects.

Our study used two groups of participants - Lean (LE) and Obese (OB) premenopausal women. The characteristics of the two groups of part at baseline (D0) and their response to the intervention are presented in Table 1. Adiposity parameters differed significantly between groups, and were associated with our inclusion criteria. In subgroups of LE and OB women (*n* = 11 for each group), MRI-based assessment of subcutaneous AT (SAT) and visceral AT (VAT) confirmed higher amounts of both SAT and VAT in participants with obesity but no difference in the SAT/VAT ratio between groups (Supplemental Table S1, Supplemental Fig. S1). The complete intervention affected parameters related to body weight similarly in both groups.

**Table 1:** Anthropometric and biochemical characteristics of the groups.

	Lean (n = 20)			Obese (n = 18)			Significance		
	Baseline	Fasting	Refeeding	Baseline	Fasting	Refeeding	Intervention phase	Adiposity	IxA interaction
*Age*	35.35 ± 7.15 (32.00–38.69)			36.46 ± 7.30 (32-83-40.09)			0.6621		
*Weight (kg)*	61.49 ± 6.67 (58.37–64.61)	59.72 ± 6.59*†††* (56.63–62.80)	60.63 ± 6.46*†††* (57.60–63.65) ###	95.37 ± 16.8 (86.97–103.80) ∗∗∗	92.67 ± 16.84*†††* (84.30–101.00) ∗∗∗	93.54 ± 17.12*†††* (85.03–102.10) ### ∗∗∗	**<0.0001**	**<0.0001**	0.1636
*BMI (kg/m*^*2*^*)*	21.49 ± 1.89 20.60–22.37	20.87 ± 1.94*†††* (19.96–21.78)	21.19 ± 1.85 *†††* (20.32–22.05) ###	33.70 ± 4.40 (31.51–35.89) ∗∗∗	32.74 ± 4.41*†††* (30.55–34.93) ∗∗∗	33.05 ± 4.50*†††* (30.81–35.28) ### ∗∗∗	**<0.0001**	**<0.0001**	0.1613
FM % (BIA)	27.15 ± 3.62 (25.45–28.85)	28.22 ± 4.04 (26.33–30.10)	26.32 ± 4.34 (24.28–28.35) ###	42.33 ± 4.74 (39.97–44.68) ∗∗∗	43.39 ± 4.72*††* (41.04–45.73) ∗∗∗	41.99 ± 4.83 (39.59–44.39) ### ∗∗∗	**<0.0001**	**<0.0001**	0.5884
FM % (DEXA)	28.73 ± 4.01 (26.85–30.61)			43.61 ± 4.39 (41.43–45.79) ∗∗∗			**<0.0001**		
*FM (kg)*	16.84 ± 3.48 (15.21–18.47)	16.98 ± 3.58 (15.31–18.65)	16.12 ± 3.80 (14.34–17.89) ##	41.08 ± 12.29 (34.97–47.19) ∗∗∗	40.99 ± 12.49 (34.78–47.21) ∗∗∗	40.07 ± 12.35*††* (33.93–46.21) ## ∗∗∗	**0.0004**	**<0.0001**	0.2745
FFM (kg)	44.76 ± 4.19 (42.79–46.72)	42.85 ± 4.35*†††* (40.81–44.88)	44.61 ± 4.22 (42.63–46.58) ###	54.41 ± 5.32 (51.79–57.06) ∗∗∗	51.89 ± 4.84*†††* (49.49–54.30) ∗∗∗	53.69 ± 5.30*†* (51.06–56.33) ### ∗∗∗	**<0.0001**	**<0.0001**	0.3126
TBW (L)	32.76 ± 3.08 (31.32–34.20)	31.36 ± 3.18 *†††* (29.87–32.85)	32.66 ± 3.09 (31.21–34.11) ###	39.83 ± 3.89 (37.90–41.77) ∗∗∗	37.99 ± 3.55 *†††* (36.23–39.76) ∗∗∗	39.31 ± 3.88 *†* (37.37–41.24) ### ∗∗∗	**<0.0001**	**<0.0001**	0.3074
*Waist (cm)*	69.43 ± 5.00 (67.08–71.77)	68.60 ± 4.89 (66.31–70.89)	70.13 ± 4.91 (67.83–72.42)	96.50 ± 10.48 (91.29–101.70) ∗∗∗	96.00 ± 11.68 (90.19–101.8) ∗∗∗	95.39 ± 10.31 (90.26–100.5) ∗∗∗	0.3557	**<0.0001**	0.1432
Hip (cm)	91.90 ± 5.32 (89.41–94.39)	93.05 ± 5.57 (90.44–95.66)	94.15 ± 5.84 (91.42–96.88)	115.20 ± 9.48 (110.50–119.90) ∗∗∗	115.20 ± 11.99 (109.30–121.20) ∗∗∗	115.40 ± 11.82 (109.50–121.30) ∗∗∗	0.2120	**<0.0001**	0.3456
Waist hip ratio	0.76 ± 0.04 (0.74–0.78)	0.74 ± 0.05 (0.72–0.76)	0.75 ± 0.4 (0.73–0.77)	0.84 ± 0.07 (0.80–0.87) ∗∗∗	0.84 ± 0.08 (0.80–0.87) ∗∗∗	0.83 ± 0.07 (0.79–0.86) ∗∗∗	0.2306	**<0.0001**	0.4620
Total cholesterol (mmol/L)	4.20 ± 0.84 (3.79–4.60)	4.47 ± 0.79 *††* (4.10–4.84)	3.79 ± 0.73 *†††* (3.44–4.13) ###	4.40 ± 0.69 (4.06–4.74)	4.64 ± 0.91 (4.19–5.10)	4.01 ± 0.76 *†* (3.64–4.39) ###	**<0.0001**	0.4715	0.8403
*HDL (mmol/L)*	1.63 ± 0.29 (1.49–1.77)	1.59 ± 0.32 (1.44–1.74)	1.52 ± 0.29 *†* (1.39–1.65)	1.21 ± 0.41 (1.00–1.41) ∗∗	1.14 ± 0.37 (0.96–1.33) ∗∗∗	1.05 ± 0.34 *†††* (0.88–1.22) ### ∗∗∗	**<0.0001**	**<0.0001**	0.0658
LDL (mmol/L)	2.23 ± 0.68 (1.91–2.54)	2.39 ± 0.62 (2.10–2.68)	1.85 ± 0.52 *†††* (1.60–2.09) ###	2.58 ± 0.73 (2.22–3.00)	2.84 ± 0.89 (2.40–3.28)	2.25 ± 0.71*†* (1.90–2.61) ###	**<0.0001**	0.0623	0.7782
TAG (mmol/L)	0.73 ± 0.25 (0.61–0.84)	1.10 ± 0.36 *†††* (0.93–1.27)	0.94 ± 0.30 *††* (0.80–1.08)	1.35 ± 0.46 (1.12–1.58) ∗∗∗	1.46 ± 0.58 (1.18–1.75)	1.57 ± 0.58 *†* (1.28–1.86) ∗∗	**<0.0001**	**0.0002**	**0.0189**

BIA. Bioimpedance; BMI. Body Mass Index in kg/m2; DEXA. dual-energy X-ray absorptiometry; FM. Fat Mass in kg; % FM. Fat Mass in percentage of total Body Mass; FFM. Fat Free Mass in kg; TBW. Total body water in kg. Values are mean ± STDEV (lower 95 % confidence interval of the mean and upper 95 % confidence interval of the mean). Variables in italics were log-transformed for the statistical tests. T-test or two-way ANOVA with post-hoc tests were used to analyze the data as appropriate. Significance was accepted if p < 0.05; ∗ refers to significant differences compared to lean (∗p < 0.05. ∗∗p < 0.01. ∗∗∗p < 0.001); † refers to significant differences compared to D0 (†p < 0.05. ††p < 0.01. †††p < 0.001); # refers to significant differences compared to D3 (#p < 0.05. ##p < 0.01. ###p < 0.001).

Blood lipids responded to the intervention irrespective of adiposity except for triglycerides (TAG), which peaked in LE women at the end of the fasting period (D3), while in OB women, TAG increased after refeeding (D5).

### 2.2 Lipolysis in AT and its regulation by insulin

AT lipolysis is maximal during fasting and suppressed during feeding, but the changes in its dynamics in obesity are not fully understood. Here, markers of lipolysis, glycerol, and FFA were measured in AT using two approaches: (1) in media conditioned by AT explants and (2) lipidomic analysis of AT.

**Fig. 1:**
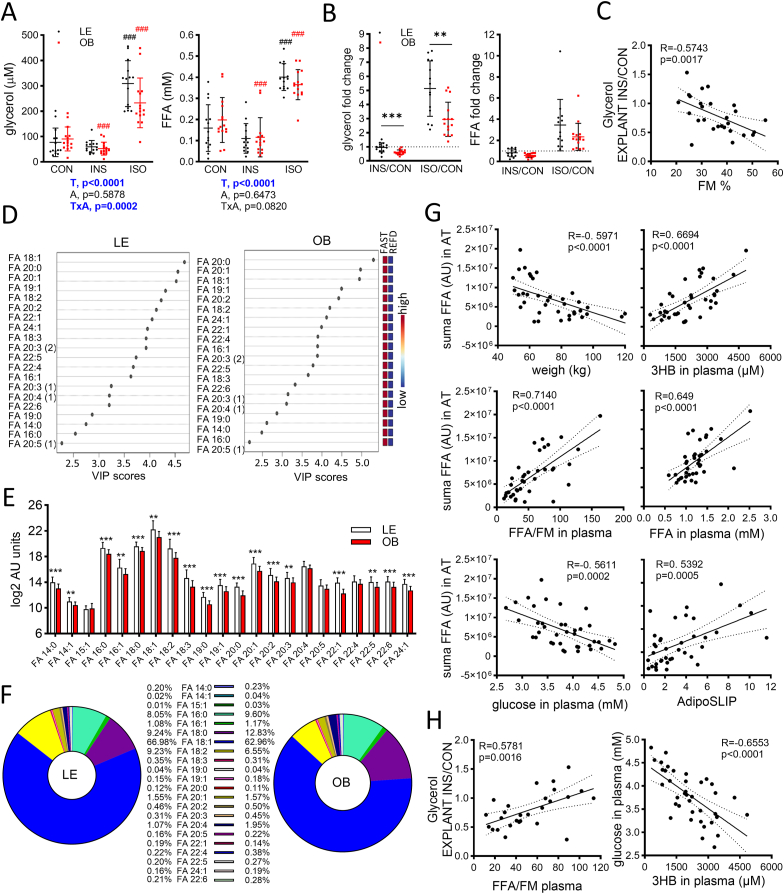
Adipose tissue and lipolysis analyses. A. FFA and glycerol levels in media conditioned by AT explants. CON, control treatment; INS, insulin; ISO, isoproterenol. Data are expressed as individual points and mean ± SD; Two-way ANOVA-Mixed model, T-effect of treatment, A-effect of adiposity, TxA-interaction between the factors; posthoc testing: difference from control conditions ^###^p < 0.001. B. Fold changes of FFA or glycerol calculated as the ratio of levels between insulin treatment and control conditions, and isoproterenol treatment and control condition, resp. in media conditioned by AT explants. Data are expressed as individual points and mean ± SD; Mann Whitney tests: difference between groups ∗∗p < 0.01, ∗∗∗p < 0.001. C. Spearman correlation between insulin suppression of unstimulated lipolysis in AT explants (INS/CON) and % fat mass and FFA in blood corrected by fat mass in kg. D. VIP score of most differing lipid molecules in fasting vs. refeeding (lipidomic analysis). E. Comparison of FFA content in AT from LE and OB women in fasted state (lipidomic analysis). Data are expressed as log2 AU mean ± SD; multiple *t*-test, FDR set up to 1 %, ∗∗p < 0.01, ∗∗∗p < 0.001. F. The relative abundance of each free fatty acid (related to the sum of all FFA) in fasted AT. G. Pearson correlations of FFA content in fasted AT with other variables in fasted state. H. Pearson correlation of 3HB with glucose in fasting plasma.

Unstimulated lipolysis in AT explants was not affected by adiposity. However, the relative increase of glycerol upon isoproterenol stimulation was greater, whereas the efficacy of 1 nM insulin to suppress glycerol release was lower in AT explants from LE compared to OB women (Fig. 1A and B). Similar trends were observed for FFA (Fig. 1A and B). Insulin-induced suppression ((INS/control ratio) of glycerol release correlated negatively with % fat mass (FM) (Fig. 1C).

Among the 384 detected lipids, FFA assessed using untargeted lipidomic analysis of AT were identified as the top 20 molecules most able to discriminate between fasted and refed states in both LE and OB women (Fig. 1D). The content of most FFA species, including the most abundant oleate (FA 18:1), was higher in LE AT compared to OB AT (D3) (Fig. 1E and F). This difference completely disappeared in the refed state (not shown). FFA content in fasted AT correlated negatively with weight and circulating glucose (Fig. 1G).

**Fig. 2:**
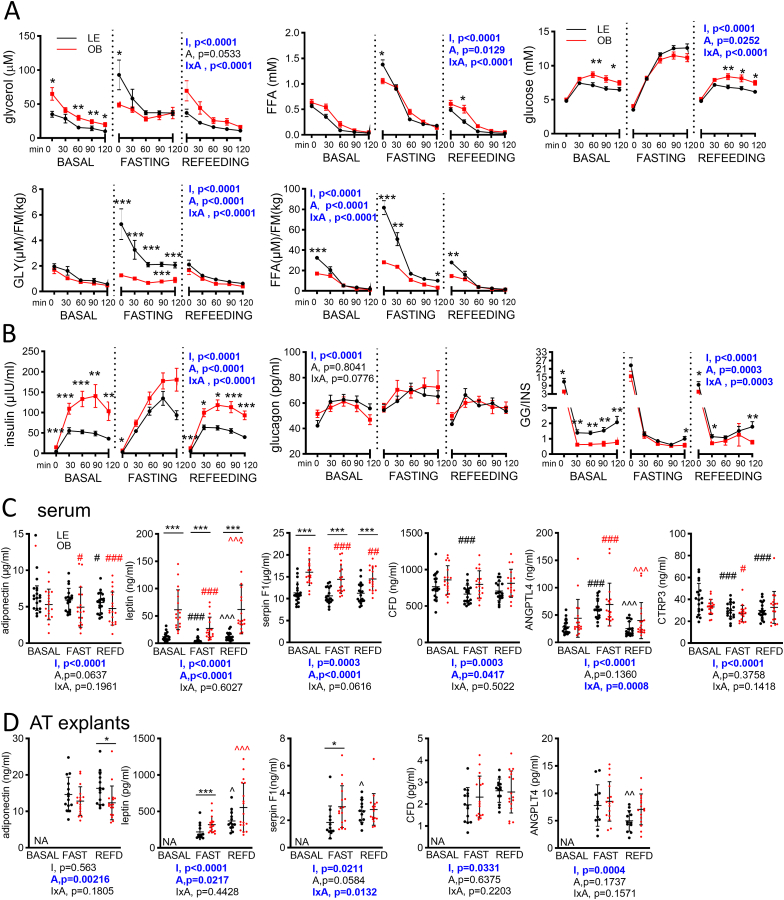
Blood profiles of metabolites and hormones during the intervention and OGTT and cytokines in serum and medium conditioned by AT explants. A. Blood FFA and glycerol levels (absolute and adjusted to fat mass in kg). B. Blood glucose, insulin and glucagon levels. Data are expressed as mean ± SD; Two-way ANOVA, I-effect of intervention, A-effect of adiposity, IxA-interaction between the factors; posthoc testing: difference between the groups ∗p < 0.05, ∗∗p < 0.01, ∗∗∗p < 0.001. C. Cytokines in serum. D. Cytokines in medium conditioned by AT explants. Data are expressed as individual points and mean ± SD; Two-way ANOVA or Mixed model, I-effect of intervention, A-effect of adiposity, IxA-interaction between the factors; posthoc testing: difference between groups ∗p < 0.05, ∗∗p < 0.01,∗∗∗p < 0.001, difference from basal time point ^#^p < 0.05, ^##^p < 0.01,^###^p < 0.001, difference from fasting time point ^^^p < 0.05, ^^^^p < 0.01,^^^^^p < 0.001.

FFA and glycerol in blood were also monitored; although they do not originate solely from AT lipolysis, their levels reflect an overall balance between production and clearance. Baseline FFA levels were the same in both groups (Table 2, Fig. 2A) and did not correlate with body weight (Pearson R = 0.131, p = 0.429). Fasting increased FFA levels in both groups, more so in LE women, and these fasting levels were inversely correlated with body weight (Pearson R = −0.556, p = 0.0003). Since post-absorptive FFA are predominantly derived from fat, we corrected the values for FM to show FFA released per kg of AT. FFA/FM levels were lower in women with obesity and correlated with weight during all intervention phases (Pearson R = 0.55–0.796, p < 0.001). Remarkably, FFA/FM levels after the 60-h fast correlated with insulin-related suppression (INS/CON ratio) of glycerol release in AT explants (Fig. 1H). Systemic glycerol levels were lower at baseline in LE vs. OB women; they increased with fasting and returned to baseline after refeeding in LE women while remaining stable throughout the intervention in OB women. Fasting glycerol levels normalized to FM were thus almost three times higher in LE than in OB women (Table 2, Fig. 2A).

**Table 2:** Metabolites, insulin, glucagon and indexes of whole body and tissue-specific insulin sensitivity or resistance.

	Lean (n = 20)			Obese (n = 18)			Significance		
	Baseline	Fasting	Refeeding	Baseline	Fasting	Refeeding	Intervention	Adiposity	IxA interaction
*Glucose (mM)*	4.82 ± 0.27 (4.69–4.94)	3.53 ± 0.46*†††* (3.31–3.74)	4.80 ± 0.30 (4.66–4.95) ###	5.04 ± 0.29 (4.89–5.18) ∗	4.08 ± 0.45*†††* (3.85–4.30) ∗∗	5.13 ± 0.26 (5.00–5.26) ### ∗∗∗	**<0.0001**	**<0.0001**	**0.0043**
*Insulin (μIU/ml)*	4.96 ± 2.28 (3.89–6.03)	3.85 ± 2.04 (2.89–4.80)	6.15 ± 2.67 (4.92–7.40) ##	16.40 ± 11.50 (10.70–22.1) ∗∗∗	8.59 ± 5.83*†††* (5.69–11.50) ∗∗	14.70 ± 5.95 (11.70–17.70) ### ∗∗∗	**<0.0001**	**<0.0001**	0.057
*Insulin/body mass (kg)*	0.077 ± 0.035 (0.061–0.094)	0.055 ± 0.034*††* (0.039–0.071)	0.093 ± 0.038 (0.075–0.111) ###	0.158 ± 0.110 (0.103–0.212) ∗∗∗	0.0741 ± 0.049*†††* (0.050–0.098) ∗∗	0.134 ± 0.048 (0.110–0.158) ### ∗∗∗	**<0.0001**	**<0.0001**	0.1648
*Glucagon (pg/ml)*	42.23 ± 18.04 (33.53–50.93)	54.47 ± 19.93*†* (44.87–64.08)	43.44 ± 15.18 (36.34–50.54) #	51.54 ± 12.37 (45.39–57.69)	56.42 ± 14.29 (49.07–63.76)	49.93 ± 10.86 (44.53–53.33)	**0.0016**	0.0705	0.2773
*Glucagon/insulin*	11.86 ± 10.34 (6.88–16.84)	23.62 ± 23.16*††* (12.45–34.78)	9.64 ± 9.87 (4.88–14.39) ###	5.04 ± 3.11 (3.54–6.54) ∗∗	15.69 ± 15.21 *†††* (49.07–63.76)	4.98 ± 2.60 (3.72–6.23) ### ∗	**<0.0001**	**0.0033**	0.3863
OGTT AUC glucose	877 ± 133 (815–939)	1270 ± 136*†††* (1206–1334)	857 ± 108 (806–907) ###	1012 ± 151 (937–1087) ∗	1202 ± 183 *†††* (1111–1293)	1001 ± 157 (923–1079) ### ∗∗	**<0.0001**	0.0853	**<0.0001**
*OGTT AUC insulin*	5401 ± 2277 (4335–6467)	10304 ± 4155*†††* (8359–12249)	6217 ± 2032 (5266–7167) ###	13112 ± 8796 (8873–17352) ∗∗∗	14178 ± 6341 (11122–17235)	11415 ± 4820 (9091–13738) ∗∗∗	**<0.0001**	**<0.0001**	**0.0018**
*HOMA-IR*	1.01 ± 0.45 (0.80–1.22)	0.52 ± 0.33*†††* (0.37–0.68)	1.22 ± 0.55 (0.96–1.48) ###	3.30 ± 2.25 (2.18–4.42) ∗∗∗	1.32 ± 0.93*†††* (0.85–1.78) ∗∗	2.94 ± 1.33 (2.28–3.60) ### ∗∗∗	**<0.0001**	**<0.0001**	0.3636
*Matsuda IS*	8.49 ± 3.15 (7.02–9.97)	7.51 ± 4.05 (5.62–9.41)	7.28 ± 3.02 (5.86–8.70)	3.28 ± 1.98 (2.29–4.26) ∗∗∗	4.35 ± 2.63*†* (3.04–5.66) ∗∗	3.34 ± 1.84 (2.43–4.26) ∗∗∗	0.2252	**<0.0001**	**0.0048**
*AdipoIR*	2.84 ± 1.83 (1.98–3.70)	5.31 ± 3.04*†* (3.91–6.72)	2.82 ± 1.39 (2.17–3.47) ##	9.46 ± 7.08 (5.94–12.98) ∗∗∗	8.63 ± 5.32 (5.99–11.28)	9.09 ± 6.03 (6.09–12.09) ∗∗∗	**0.0293**	**<0.0001**	**0.0087**
*AdipoSLIP*	2.60 ± 1.70 (1.73–3.33)	5.27 ± 3.17*†* (3.78–6.75)	2.69 ± 1.42 (2.02–3.35) ##	2.55 ± 1.95 (1.57–3.52)	2.29 ± 1.45 (1.57–30.1) ∗∗	2.26 ± 1.34 (1.60–2.93)	**0.0185**	**0.0293**	**0.0087**
*GLY (μM)*	34.97 ± 19.98 (25.62–44.32)	92.90 ± 97.53 *†††* (47.26–138.50)	37.18 ± 25.19 (25.39–48.96) ###	64.95 ± 38.94 (44.93–84.97) ∗∗	49.32 ± 15.22 (41.49–57.14)	69.63 ± 61.15 (38.19–101.1)	**0.0027**	0.1064	**0.0003**
*GLY (μM)/FM (kg)*	1.99 ± 1.6 (1.50–2.49)	5.46 ± 5.59*†* (2.76–7.78	2.12 ± 1.56 (1.41–2.82) #	2.45 ± 3.41 (0.76–4.15)	1.55 ± 1.32 (0.90–2.21) ∗∗∗	1.64 ± 1.43 (0.92–2.35)	**0.0027**	**0.0011**	**0.0003**
*FFA (mM)*	0.56 ± 0.20 (0.47–0.65)	1.38 ± 0.41*†††* (1.19–1.57)	0.49 ± 0.20 (0.39–0.58) ###	0.63 ± 0.26 (0.51–0.76)	1.06 ± 0.23*†††* (0.94–1.17) ∗	0.61 ± 0.30 (0.46–0.76) ###	**<0.0001**	0.9979	**0.0412**
*FFA (μM)/FM (kg)*	32.38 ± 11.86 (26.83–37.93)	81.61 ± 30.80*†††* (67.20–96.03)	27.93 ± 11.56 (22.52–33.34) ###	16.62 ± 8.61 (12.24–20.80) ∗∗∗	27.50 ± 10.00*†††* (22.53–32.47) ∗∗∗	14.85 ± 7.02 (11.36–18.33)### ∗∗∗	**<0.0001**	**<0.0001**	**<0.0001**
*FFA μM/FFM (kg)*	12.81 ± 4.134 (10.88–14.74)	32.54 ± 12.08*†††* (26.89–38.19)	11.40 ± 5.11 (9.01–13.79) ###	12.28 ± 5.47 (9.56–15.00)	20.58 ± 5.60*†††* (17.80–23.37) ∗∗∗	11.54 ± 5.42 (8.85–14.24)###	**0.0796**	**<0.0001**	**0.0419**
*3BH (μM)*	84.65 ± 59.27 (59.61–112.40)	2819.00 ± 875.42*†††* (2409–3229)	29.20 ± 11.27*†††* (23.88–34.52) ###	51.22 ± 26.70 (37.94–64.50)	1416.22 ± 740.78*†††* (1048–1785) ∗∗∗	28.11 ± 12.29*††* (22.00–34.22)###	**<0.0001**	**0.0002**	**0.0099**
*lactate (μM)*	986.55 ± 227.04 (880.3–1093)	1204.90 ± 245.47*††* (1090–1320)	1756.70 ± 468.28*†††* (1538–1976)###	1387.17 ± 420.20 (1178–1596) ∗∗	1253.78 ± 282.52 (1113–1394)	1891.89 ± 439.13*††* (1674–2110)###	**<0.0001**	**0.0244**	**0.0073**
valine (μM)	198.60 ± 26.86 (186–211.2)	398.15 ± 48.28*†††* (375.6–420.7)	169.30 ± 20.60*†††* (159.7–178.9)###	226.17 ± 43.51 (204.5–247.8)	360.22 ± 640.65*†††* (340–380.4) ∗	202.06 ± 28.10*†* (188.1–216.0)### ∗∗	**<0.0001**	0.0749	**<0.0001**

AdipoIR, Adipose tissue insulin resistance index; AdipoSLIP, index of suppression of AT lipolytic activity by fasting levels of insulin; GLY, Glycerol; FFA, Free fatty acids; 3BH, 3-hybroxybutyrate; HIRI, hepatic insulin resistance index. Values are mean ± STDEV and lower 95 % confidence interval of the mean and upper 95 % confidence interval of the mean. Variables in italics were log-transformed for the statistical tests; *t*-test or two-way ANOVA with post-hoc tests were used to analyze the data as appropriate. Significance was accepted if p < 0.05; ∗ refers to significant differences compared to lean (∗-p<0.05. ∗∗p < 0.01. ∗∗∗p < 0.001; † refers to significant differences compared to D0; # refers to significant differences compared to D3.

### 2.3 Circulating 3-hydroxybutyrate, lactate, leucine, and valine at baseline and in response to the intervention

In addition to lipolysis, fasting increases ketogenesis (reflected by 3- hydroxybutyrate levels (3HB), lactate production, and proteolysis (reflected by leucine and valine levels).

3HB levels were similar in both groups at baseline; they dramatically increased in response to fasting, and decreased below baseline after refeeding (Table 2). The fasting-induced increase in 3HB was more pronounced in LE women. Circulating levels of 3HB positively correlated with FFA content in AT and negatively with glucose (Fig. 1G and H). Lactate, an important gluconeogenic substrate and signaling molecule also produced by AT [13], increased after fasting, but only in LE women and after refeeding in both groups. Two analyzed branched chain amino acids (BCAA), leucine and valine, also increased in response to fasting and returned to or below baseline after refeeding (Table 2).

### 2.4 Glucose, insulin, glucagon, and indexes reflecting sensitivity to insulin at baseline and in response to the intervention

Basal insulin and glucose levels were lower in LE compared with OB women (Table 2). Since basal insulin levels correlated with body mass (Pearson R = 0.582, p = 0.0001), we corrected insulin levels to body weight, but the difference between groups persisted. Fasting reduced blood glucose levels, more so in LE women (rel. delta D3-D0 glucose: LE -26.6 ± 8.3 %; OB -18.6 ± 11.5 %, p = 0.0161). Insulin levels only dropped in OB women; however, when adjusted for body mass, the reduction was noticeable in both groups. After refeeding, glucose and insulin levels returned to baseline (Table 2).

Baseline glucose tolerance (OGTT-AUC glucose) was superior in LE compared to OB women; however, it worsened during fasting in both groups, especially in LE women, ultimately becoming equal to levels seen in OB women (Table 2, Fig. 2A, relative delta D3-D0, LE 46.9 ± 21.0 %; OB 20.4 ± 17.8 %, p = 0.0002). Baseline OGTT-AUC insulin was higher in OB women but the differences between the groups disappeared during fasting due to relatively higher fasting-induced change in LE vs. OB women (Table 2, Fig. 2B, relative delta D3-D0, LE 102.6 ± 88.5 %, OB 29.1 ± 65.02 %, p = 0.0065). Glucagon levels, a key insulin-counter-regulatory hormone, were similar in both groups throughout the intervention. Fasting increased the glucagon-to-insulin ratio in both groups (Table 2). Throughout the intervention, oral glucose intake reduced glycerol and FFA levels in both groups (Fig. 2A).

Insulin sensitivity/resistance indices of the body and AT were calculated. OB women had a lower Matsuda index and higher HOMA-IR than LE women throughout the intervention (Table 2). HOMA-IR decreased in response to fasting (due to a reduction in both insulin and glucose levels) and returned to baseline after refeeding in both groups; the Matsuda index remained unchanged in LE women throughout the intervention and peaked after fasting in OB women.

The AdipoIR index, estimating *in vivo* AT insulin resistance, was about three times higher in OB than LE women at baseline and refeeding phases. After fasting, it increased in LE to levels observed in OB women. Contrary to expectations, AdipoIR was not positively correlated with fasted AT FFA levels (Pearson R = −0.206, p = 0.215). This prompted us to adjust the AdipoIR index to fat mass and body mass, so for our calculations, we used FFA levels normalized to FM and insulin levels normalized to body mass (kg). The resulting index, which we termed AdipoSLIP (adipose tissue suppression of lipolysis), correlated well with FFA content in AT, showing higher levels in LE compared to OB women after a 60-h fast (Fig. 1G, Table 2).

### 2.5 Metabolic rate and substrate utilization *in vivo*

Resting metabolic rate (RMR) adjusted to fat free mass (FFM) (kg) was not affected by fasting but decreased in response to refeeding irrespective of group (Supplemental Table S2). The respiratory quotient (RQ), which provides an estimate of preferred substrate oxidation, decreased during fasting and returned to baseline after refeeding. Neither the RQ changes nor the relative increase in RQ in response to glucose ingestion were affected by adiposity (Supplemental Table S2).

### 2.6 Adipokines in circulation and produced by AT *ex vivo*

Recognizing that cytokines may affect lipolysis and insulin sensitivity, we analyzed several adipokines previously implicated in the regulation of these processes during fasting [[14], [15], [16], [17]], both in the blood and in a medium conditioned by AT explants. In the blood, leptin, serpin F1, and complement factor D (CFD) but not adiponectin, CTRP3 (Complement C1q Tumor Necrosis Factor-Related Protein 3), and ANGPTL4 (Angiopoietin Like 4) levels were dependent on adiposity (Fig. 2C). Although fasting and refeeding affected levels of all monitored cytokines, the response to the intervention differed between LE and OB women only for ANGPTL4 and non-significantly for serpin F1.

*Ex vivo*, adiposity affected AT secretion of adiponectin and leptin, with a trend for serpin F1 (Fig. 2D). Leptin secretion increased, and ANGPTL4 decreased after refeeding in both groups, which was similar to what we saw in blood. Serpin F1 was the only adipokine with a differential response to the intervention between LE and OB women. Still, levels of leptin and serpin produced by fasted AT correlated with each other (Pearson, R = 0.634, p = 0.0002) and also with their respective blood levels (leptin, R = 0.436, p = 0.018; serpin, R = 0.541, p = 0.003). Interestingly, both fasting blood levels and AT secretion of serpin F1 correlated with TAG levels (Pearson, R = 0.630 and 0.625, both p < 0.0001). CFD secretion had a similar pattern to serpin F1, though the refeeding-induced increase in LE women was not significant.

## 3 Discussion

Our model of a 60-h fast and subsequent refeeding in lean and obese women aimed to answer how excessive adiposity affects adaptation to fasting and subsequent refeeding, namely AT function and the development of acute IR. Although many studies have examined AT lipolysis during fasting, models that combine fasting with refeeding have rarely been explored in humans, as mentioned in a recent review [18]. Therefore, our results offer unique insights into AT adaptations to these physiological challenges.

### 3.1 Lipolysis in AT and its regulation by insulin

Obesity-linked development of AT dysfunction includes defects in lipolytic and endocrine activity, contributing to the development of cardiometabolic diseases. However, these AT alterations may also be perceived as inevitable adaptations to changes in body composition and increased availability of energy reserves. In agreement with this perception of obesity, our data showed that adipose FFA flux was attenuated in women with obesity compared with lean women, evidenced by lower FFA levels both in circulation and within AT upon fasting in OB women. This corroborates findings by S. Klein, indicating that AT lipolytic activity decreases with increasing adiposity [[6], [7], [8]], probably to maintain non-toxic circulating FFA levels [19]. On the other hand, the reduced lipolytic response results in reduced triglyceride turnover, associated with impaired weight maintenance and accelerated aging [20]. The mechanisms underlying these adaptations of obese AT are likely very complex. These may involve a deregulation of various signaling pathways as well as structural changes. It has been described that obese AT suffers from capillary rarefaction [21] and reduced lymphatic drainage [22]. The lower density of both blood and lymphatic capillaries could significantly impact the exchange of nutrients, hormones, cytokines, enzymes, and lipolytic products in the interstitium since endothelial cells control many of these processes, including AT lipolysis [23,24]. Consequently, defective microcirculation *in vivo* could contribute to altered insulin and lipolytic signaling within the obese AT microenvironment. Notably, a new class of anti-obesity drugs, glucagon-like peptide-1 receptor agonists (GLP-1 RAs), have been demonstrated to directly improve endothelial function [25]. It is, therefore, conceivable that through this mechanism, GLP-1 RAs could help normalize the regulation of FFA flux within AT. However, further research is required to confirm this hypothesis.

In addition, data from AT explants suggested that fasted AT from OB women was more sensitive to insulin than AT from lean women. AT insulin sensitivity can be estimated from blood levels of FFA and insulin by calculating the AdipoIR index. However, the AdipoIR index is calculated as a product of FFA and insulin levels, without compensating for body composition, which affects both variables [26], which Sondergaard et Jensen pointed out as an obvious bias [27]. This led us to define a new index, which we called AdipoSLIP. AdipoSLIP adjusts FFA concentration for fat mass, and insulin for body weight, rendering it independent of the overall adiposity. The validity of AdipoSLIP is based on observed correlation of AdipoSLIP (but not the AdipoIR index) with FFA levels in AT reflecting lipid mobilization. In addition, the systemic lipolytic response of OB women was blunted despite the more pronounced fasting-related drop in insulin, which drives post-digestive lipolysis [8]; further the observed decrease in insulin levels (delta D3-D0) did not correlate with increased FFA release (Pearson, R = −0.206, p = 0.219). Therefore, based on both *in vivo* and *ex vivo* data, it appears that AT insulin resistance after fasting is lower in women with obesity. This observation is reflected more accurately by the AdipoSLIP index than AdipoIR.

Another marker of lipolysis is glycerol, whose levels did not change in response to fasting in OB women. Since glycerol, together with lactate, are the primary gluconeogenic substrates produced by AT [28,29], we could speculate that after glycogen depletion, OB women increase glycerol and lactate utilization to ensure gluconeogenesis [30].

### 3.2 Circulating 3-hydroxybutyrate, lactate, leucine, and valine at baseline and in response to the intervention

3HB levels were dramatically induced by fasting but to a lesser extent in OB women. The observed strong correlation between FFA content in AT and circulating levels of 3HB suggests that 3HB could be a surrogate marker of AT lipolysis. It indicates that FFA produced by AT are indeed the major source for liver-produced ketone bodies, contributing to the close interplay between AT and liver metabolism during fasting [31]. Lower fasting FFA levels may therefore explain lower 3HB levels in OB women who rely more on endogenous glucose production [30]. Accordingly, we observed a negative relationship between glucose and 3HB levels during fasting. Interestingly, lower levels of 3HB were observed in overweight/obese subjects who were resistant to low-calorie diet weight loss and thus appeared to be more metabolically adapted to preserve their levels of adiposity [32]. This supports a connection between the lower inducibility of lipolysis and resistance to caloric restriction, potentially hindering the intentional efforts of women with obesity to lose weight.

Adipocytes/AT produce a substantial amount of lactate, and people with obesity have been reported to have higher lactate levels [33]. We observed, both in LE and OB women, the same impact of adiposity on lactate at baseline, as well as similar increase in lactate in response to refeeding. This fits with the observation that adipocytes maintain high lactate production post-refeeding to restore hepatic glycogen levels [34].

Prolonged fasting inevitably affects lean body mass, which provides amino acids to support vital protein synthesis and gluconeogenesis, and the fasting levels of BCAA primarily reflect the breakdown of muscle protein. BCAA levels are also affected by obesity since AT is considered a major contributor to whole-body BCAA metabolism [35]. During a 60-h fast, BCAA levels increased more substantially in lean women, implying they have either higher muscle breakdown or lower transamination of BCAA in AT, the latter being linked with higher AT IR. This further supports the idea that fasting is associated with higher AT IR in lean women.

### 3.3 Glucose, insulin, glucagon and indexes reflecting sensitivity to insulin at baseline and in response to the intervention

Our findings on glucose dynamics and glucoregulatory factors partially align with previous research. In line with Horowitz et al., Wijngaarden et al., and Bak et al. [30,36,37], we observed a decrease in glucose levels after fasting. This was less pronounced in OB women, suggesting increased gluconeogenesis in the liver or kidney [30,38]. However, our findings did not show a notable group difference in the glucagon/insulin ratio, which is the primary regulator of gluconeogenesis, as shown by an earlier study using mice and humans [39]. Nevertheless, a fasting-induced reduction in insulin levels was only observed in OB women, possibly being prevented in LE women by higher fasting FFA levels [40].

The widely used index of whole-body IR, HOMA-IR, is calculated from blood glucose and insulin levels after overnight fasting. Our study showed that a 60-h fast resulted in lower HOMA-IR despite obviously worsened glucose tolerance. Therefore, while the HOMA-IR index is appropriate for epidemiology studies using the standard fasting lengths, it appears to be unsuitable for dynamic conditions with high concentrations of ketone bodies replacing glucose. The Matsuda index, which reflects the dynamic potential of insulin to lower glucose levels during OGTT, increased mildly after fasting but only in OB women. Thus, although glucose tolerance apparently worsened during the 60-h fast, neither group showed signs of acute IR; instead, the increased Matsuda index suggests an improvement of IS during the 60-h fast in OB women.

### 3.4 Substrate utilization *in vivo*

Chronic IR is related to diminished metabolic flexibility, i.e., the ability to utilize substrates based on their abundance, which leads to changes in RQ. Thus, RQ changes can be used as indirect indicator of metabolic flexibility. After the 60-h fast, both groups showed a decrease in RQ, suggesting a shift towards lipid oxidation, but in contrary to a study by Wijngaarden et al. [36] we observed no group differences in the baseline RQ or the ΔRQ during OGTT. Given that the obese participants in Wijngaarden's study had a higher waist-to-hip ratio indicating metabolically unhealthy obesity [41], our findings may imply that although the metabolic adaptation of OB women to fasting is less dynamic, their metabolic flexibility is similar to LE women, except in cases of metabolically unhealthy obesity.

### 3.5 Adipokines in circulation and produced by AT *ex vivo*

Since different cytokines regulate IR and metabolic function of AT [42], our study, in addition to examining the prototypical adipokines, leptin and adiponectin, both of which are crucial for weight regulation and insulin sensitivity, we also examined serpin F1 (an adipose triglyceride lipase (ATGL) coactivator [16]), CFD (a fasting-induced cytokine [14,42]), CTRP3 (a cytokine with glucose-lowering and anti-inflammatory effects [17]), and ANGPTL4 (a lipoprotein lipase inhibitor [15]). While leptin levels significantly dropped during fasting, agreeing with previous studies [36,43], adiponectin levels steadily decreased from baseline to refeeding, deviating from an earlier report [43]. Similarly, levels of CTRP3, an adiponectin paralogue, declined with fasting, and delta D3-D0 of CTRP3 and adiponectin levels correlated with each other (not shown). CFD levels also fell during fasting. Importantly, these three adipokines, i.e., adiponectin, CTRP3, and CFD, play roles in lipid opsonization and alternative complement activation [44], supporting a predicted link between the metabolic state of AT and innate immunity [45].

As documented previously, ANGPTL4 increased during fasting [15] and normalized upon refeeding, but the overall response to the intervention differed between groups, with less pronounced changes in OB women. This could be related to lower fasting FFA levels, implicated in the upregulation of ANGPTL4 expression [45]. Another adipokine regulated differentially between LE and OB women, serpin F1, correlated with plasma triglycerides. Being produced by adipocytes and hepatocytes [46], serpin F1 might thus play a significant role in the adipose-liver signaling axis for TAG metabolism via its interaction with ATGL.

## 4 Limitations

The current study has several limitations. Firstly, women in the groups were not matched for FFM, potentially obscuring the actual impact of adiposity on the variables studied. Additionally, AT biopsies were not performed at the baseline time point to avoid multiple AT biopsies from the same side of the abdomen. Only abdominal subcutaneous AT was studied. However, we acknowledge that femoral AT, and to a lesser extent visceral AT, which can contribute to systemic FFA levels, would also be affected by our intervention. We also did not use isotopes to assess *in vivo* FFA and glucose release and uptake due to the unavailability of clinically certified isotopes. This limits our ability to draw definitive conclusions about substrate fluxes. Nevertheless, our lipidomic data provided a valuable snapshot of lipolysis directly in AT, free from the biases common in physiological methods like blood flow changes, contributions from other tissues to FFA metabolism, and the inability to monitor lymphatic metabolite routing. Finally, a recent paper focused on the proteomic signature of fasting [47] showed that some adaptations require longer (7-day) fasting periods. Still many systemic changes were evident after 60 h of fasting. Therefore, even with shorter fasting periods, we were able to capture significant acute alterations in metabolic responses and effectively highlight the differential adaptations between LE and OB women. Additionally, longer fasting periods induce profound secondary adaptations related to weight loss, which were not the focus of our study.

## 5 Conclusions

In summary, despite initially higher IR in OB women, a 60-h fast reduced glucose tolerance regardless of adiposity. The fasting/refeeding challenge affected the endocrine activity of AT, although differences between groups were modest. Our findings that the lipolysis and development of acute IR in AT in response to fasting was blunted in women with obesity, suggest that AT adapts to obesity to limit FFA lipotoxicity. However, in long-term obesity, the negative aspects of this adaptation, including lower ability of individuals with obesity to lose weight during short-term energy deficits, prevail.

## 6 STAR methods text

### 6.1 Subjects

**Supplemental Figure S2:**
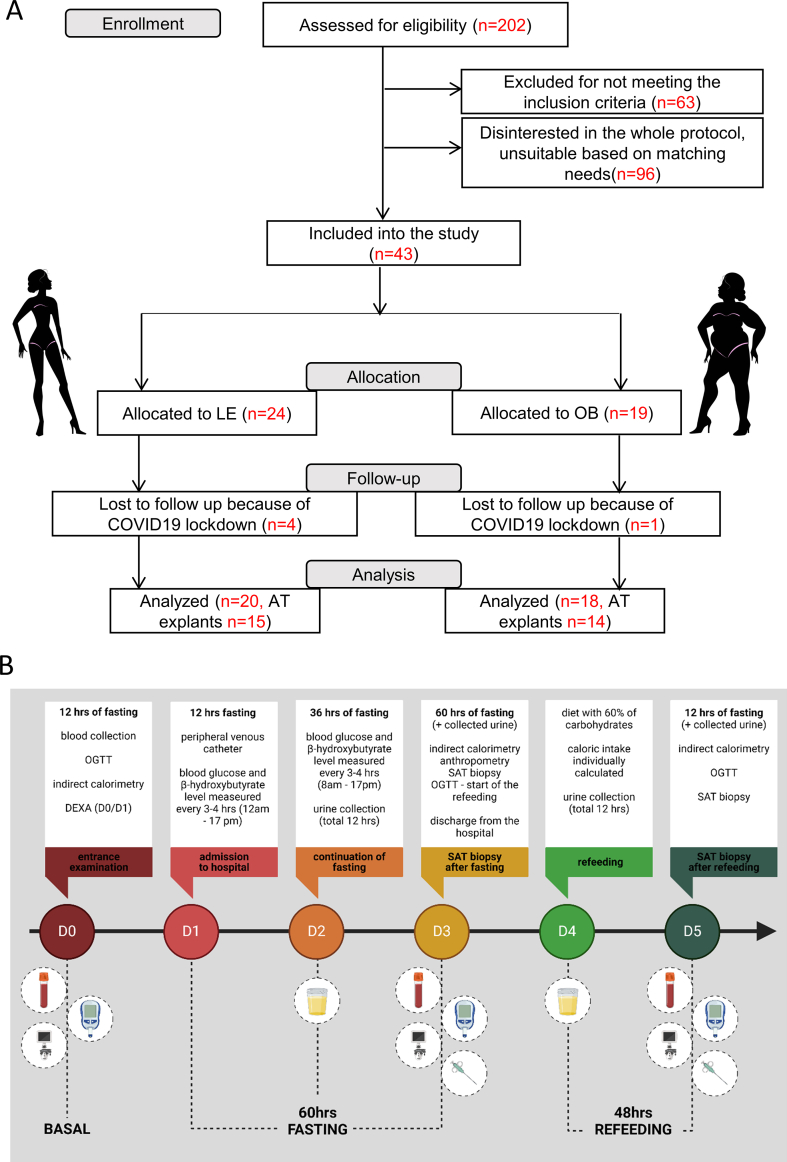
Supplemental Figure S2. Diagram of the study design, related to Table 1. A. Consort diagram of the study. B. Scheme depicting the type of investigation and sample collection during the intervention. LE, lean women; OB, women with obesity; SAT, subcutaneous adipose tissue. Created with BioRender.com.

This work is based on the clinical study DELISA (registered at https://clinicaltrials.gov/study/NCT04260542?term=NV19-01-00263&rank=1 NCT04260542) approved by the Ethical Committee of Kralovske Vinohrady University Hospital, Prague, Czech Republic (EK-VP/31/0/2018, EK-VP/31/1/2018). The study was conducted between September 2019 and July 2021 at Kralovske Vinohrady University Hospital as a single-center, non-randomized trial. All subjects gave informed consent before the start of the study. The scheme of study design is shown in Supplemental Fig. S2A. In total, 202 premenopausal Caucasian women (sex assigned at birth) were evaluated for compliance with inclusion and exclusion criteria (using an initial questionnaire and an initial screening). Only women within a narrow age range (25–45 years) were selected for the study to limit the influence of sex assigned at birth and age that undoubtedly exist in AT. The inclusion criteria were age 25–45 years and a BMI of 18–40 kg/m^2^. Exclusion criteria were diagnosed cancer, diabetes mellitus, liver and renal diseases, any major cardiovascular event, bariatric surgery, allergy to lidocaine, positive serology for hepatitis (B and C) and HIV, smoking above ten cigarettes/day, sleep apnea, poor venous status, weight-change more than 3 kg in last three months, untreated hyper- or hypothyroidism and long-term use of medication, abnormal sleep/wake patterns and shift-work. The 43 women who met the criteria were divided into two groups according to BMI: (1) lean (LE; BMI 18–25 kg/m^2^), *n* = 24; and (2) with obesity (OB; BMI 30–40 kg/m^2^), *n* = 19 and matched for age and physical activity using the IPAQ questionnaire. Five women did not finish the protocol due to the COVID-19 lockdown, so 20 LE and 18 OB participants completed the protocol.

### 6.2 Dietary intervention

**Supplemental Figure S3:**
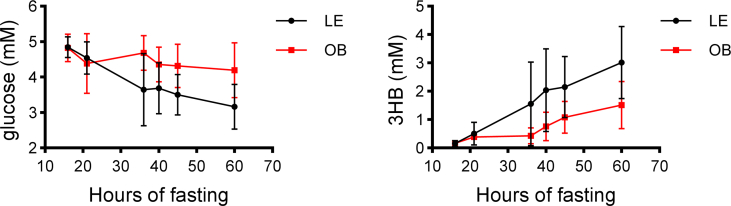
Supplemental Figure S3. Glucose and 3HB levels measured in capillary blood during hospitalization (fasting period), related to Table 1. Levels of the two metabolites were measured by handhold devices using the single use strips. Data are expressed as mean ± SD.

The intervention included a 60-h fast and a subsequent 48-h refeeding period. These time periods were selected based on expected outcomes (Fasting: depletion of liver glycogen, suppression of *de novo* lipogenesis in the liver and AT, maximum difference in insulin and glucagon production, and peak lipolytic activity; Refeeding: inhibition of lipolysis and stimulation of lipogenesis [[48], [49], [50]]) and clinical feasibility. During fasting days, subjects were hospitalized in the University Hospital Kralovske Vinohrady to monitor fasting compliance and to ensure the fast elimination of any possible severe metabolic disturbances induced by fasting. Participants were tested for 3HB levels and glucose six times during their hospitalization using capillary blood and test strips to monitor adherence to the fasting intervention (Supplemental Fig. S3). During the refeeding period, standardized meals (60 % carbohydrates, 15 % protein, and 25 % fat) were provided to meet individual daily caloric requirements calculated from the participants’ resting metabolic rate (RMR) multiplied by 1.3 to correct for physical activity.

### 6.3 Clinical investigations and AT sampling

Clinical investigations (Supplemental Fig. S2B) were performed one week before the intervention (D0), after fasting (D3), and after refeeding (D5). Anthropometric characteristics and body composition were measured using bio-impedance (Multifrequency bioimpedance instrument NutriPlus) and dual emission X-ray absorptiometry (DEXA; instrument LUNAR, only at D0). In women entering the study between June 2020 and July 2021 (*n* = 11, each group), subcutaneous (SAT) and visceral (VAT) AT was evaluated using MRI (whole-body 3T MR scanner Vida, Siemens, Germany equipped with 30-channel surface body matrix and 32-channel spine coil) at D0 (Supplemental Fig. S1). Magnetic resonance (MR) volumetry was based on T2-weighted HASTE sequences with repetition with a base resolution of 512 pixels measured while participants held their breath. SAT, VAT, and muscle were segmented manually using ITK-SNAP software from a single slice in the middle of the 3^rd^ lumbar vertebra in the transverse plane.

Respiratory quotients (RQ) were measured using indirect calorimetry (Quark RMR, Cosmed, Inc., Italy), and RMR was calculated using a modified Weir formula [51]. Glucose tolerance was assessed using oral glucose tolerance tests (OGTT): after an overnight fast, subjects drank 75 g of glucose within 5 min, and blood samples were taken at 0, 30, 60, 90, and 120 min.

Needle biopsies of SAT were performed in the abdomen, 8–10 cm lateral to the umbilicus, at D3 (after a fast) and D5 (after refeeding, 10 min after the end of OGTT, and on the contralateral side of the abdomen from the D3 sample). After disinfection, the skin and AT were anesthetized with a 1 % mesocaine solution. The skin was incised with a scalpel, and a 16G needle with a syringe was used to aspirate 1–2 g of AT, then the area was compressed and cooled. The samples of AT were washed with PBS, divided into aliquots, immediately processed for AT explants or snap frozen in liquid nitrogen, and stored at −80 °C until analysis.

### 6.4 Blood/conditioned media analyses and calculations

Blood lipids, glucose, insulin, and certain metabolites were analyzed in certified laboratories: glucose was assessed using the hexokinase reaction (KONELAB, Dreieich, Germany); insulin by using an electro-chemiluminescent enzyme immunoassay (Elecsys Insulin, ROCHE, Basel, Switzerland); total cholesterol and triglycerides using enzymatic method kits (KONELAB); high-density lipoprotein-cholesterol (HDL) was measured using polyethylene glycol-modified enzymatic assay kits (ROCHE); and low-density lipoprotein–cholesterol (LDL) was calculated using the standard Friedewald equation. Glycerol and FFA were measured using colorimetric assays (Randox, Crumlin, United Kingdom). Glycerol was also monitored at the bedside using a DietSee glycerometer [52]. 3HB, lactate, leucine, and valine were determined using capillary electrophoresis with contactless conductivity detection, as described in our previous papers [53,54]. Glucagon was measured using radio-immunoassays (Millipore, Billerica, MA, United States) and cytokine levels using ELISA DuoSets (R&D Systems, Abingdon, United Kingdom). HOMA-IR, the Matsuda index, and Adipo IR were calculated according to Gastaldelli [55].

### 6.5 Lipolysis in AT explants

Excised AT was cut into small pieces, washed two times, and incubated in Krebs/Ringer phosphate buffer (KRB, pH 7.4) supplemented with 20 g/L of FFA-free BSA and 1 g/L of glucose for 1 h at 37 °C in a shaking water bath, then washed again to remove proteins released from damaged cells. Then, 70 mg of briefly dried AT was carefully weighed and incubated in KRB supplemented with water, 1 nM insulin, or 0.1 μM isoproterenol to induce or suppress lipolysis. After 2 h at 37 °C in a shaking water bath, samples were placed on ice to stop lipolysis, the conditioned medium was collected and clarified by centrifugation, and supernatants were stored at −80 °C until analysis.

### 6.6 Lipidomic analysis

FFA profiles were analyzed using liquid chromatography/mass spectrometry (LC/MS). Extraction of AT samples (20 ± 2 mg) was performed using a biphasic solvent system of cold methanol, methyl *tert*-butyl ether (MTBE), and water [56]. The LC/MS analysis consisted of a Vanquish UHPLC System coupled to a Q Exactive Plus mass spectrometer (Thermo Fisher Scientific, Bremen, Germany). MS-DIAL v. 4.80 software was used for data processing [57], and data were normalized using locally estimated scatterplot smoothing (LOESS) followed by sample‐weight normalization [58].

### 6.7 Statistical analysis

All datasets were tested for normality or lognormality using a battery of tests (Shapiro-Wilk, D'Agostino & Pearson, Anderson-Darling, Kolmogorov-Smirnov). Non-normally distributed variables were log-transformed for analysis and back-transformed for presentation. Baseline differences between groups were analyzed using the unpaired *t*-test. Effects of fasting and refeeding were analyzed using two-way repeated measures ANOVA with a Sidak (adiposity effect) or Tukey (intervention effect) correction to account for multiple comparisons. The lipidomic dataset was processed using MetaboAnalyst [59], where data were normalized using log-transformation and Pareto scaling. A partial least-square discriminant analysis (PLS-DA) with variable importance in projection (VIP) scores calculation was performed to evaluate the differences between the groups after the fasting (D3) and refeeding (D5) periods. Correlations are expressed using Pearson's or Spearman's correlation coefficient as appropriate. Statistical analyses were performed using GraphPad Prism version 10.2.1 for Windows (GraphPad Software, La Jolla, CA, USA). Significance was set at p < 0.05, and values were expressed as mean ± standard deviation or 95 % CI.

**Table:** Key resources table

REAGENT or RESOURCE	SOURCE	IDENTIFIER
Biological samples		
Human subcutaneous adipose tissue from abdominal area (women)	University Hospital Kralovske Vinohrady, Prague, CZ	NCT04260542
Human plasma and serum (women)	University Hospital Kralovske Vinohrady, Prague, CZ	NCT04260542
Chemicals, peptides, and recombinant proteins		
Mesocain (10 mg/ml)	Zentiva	N/A
PBS (10×) w/o Ca&Mg	Lonza	Cat# BE17-515Q
Krebs/Ringer phosphate buffer	University Hospital Pharmacy Kralovske Vinohrady	N/A
FFA-free BSA	Sigma-Aldrich	Cat# A7030-50G
Glucose (OGTT) 75 g	University Hospital Pharmacy Kralovske Vinohrady	N/A
Insulin solution human	Sigma-Aldrich	Cat# 19278-5 ML
Isoproterenol hydrochloride	Sigma-Aldrich	Cat# 16504-500 MG
Critical commercial assays		
FFA kit	RANDOX	Cat# FA115
GLYCEROL kit	RANDOX	Cat# GY105
Glycerometer	DietSee	https://pubmed.ncbi.nlm.nih.gov/33766325/^41^
Glucagon RIA Kit	Millipore	Cat# GL-32K
Human leptin ELISA DuoSet Kit	R&D systems Biotechne	Cat# DY398
Human adiponectin ELISA DuoSet Kit	R&D systems Biotechne	Cat# DY1065
Human serpin F1 (PEDF) ELISA DuoSet Kit	R&D systems Biotechne	Cat# DY1177-05
Human complement Factor D (CFD) ELISA DuoSet Kit	R&D systems Biotechne	Cat# DY1824
Human CTRP3 ELISA DuoSet Kit	R&D systems Biotechne	Cat# DY7925-05
Human ANGPTL4 ELISA DuoSet Kit	R&D systems Biotechne	Cat# DY3829
Deposited data		
Lipidomic data	This paper	https://doi.org/10.5281/zenodo.13771077
Software and algorithms		
GraphPad Prism 10.2.1	GraphPad Software	https://www.graphpad.com/
MetaboAnalyst 6.0	JavaServer Faces Technology, PrimeFaces library (v13.0)	https://www.metaboanalyst.ca/MetaboAnalyst/home.xhtml^46^
Other		
Lipid spectrum and basic metabolomics analysis (multiplatform LC-MS-based approach LIMeX-5D) commercial analysis	Institute of Physiology CAS, Prague, CZ this paper	https://pubmed.ncbi.nlm.nih.gov/33572810/^47^
Blood lipids and glucose commercial analysis	Department of Laboratory Diagnostics, University Hospital Kralovske Vinohrady, Prague, CZ this paper	https://www.fnkv.cz/centralni-laboratore-uvod.php
Insulin level in serum commercial analysis	SPADIA, Prague, CZ this paper	https://www.spadia.cz/en/
3-hydroxybutyrate and L-lactate analysis	this paper	https://pubmed.ncbi.nlm.nih.gov/35636371/^42^
L-leucine and L-valine analysis	this paper	https://pubmed.ncbi.nlm.nih.gov/34228371/^43^

**Resource and data availability**

**Lead contact**

Further information and requests for datasets and protocols should be directed to and will be fulfilled by the Lead Contact, Lenka Rossmeislova (lenka.rossmeislova@lf3.cuni.cz).

**Materials availability**

This study did not generate new unique reagents.

**Data and code availability**

The explanation of codes used in lipidomic dataset deposited at Zenodo repository (https://zenodo.org/) will be available from the Lead Contact upon reasonable request and after the permission of the Ethical Committee of Kralovske Vinohrady University Hospital, Prague, Czech Republic.

This study does not report original code.

## Funding statement

The study was supported by the 10.13039/501100003243Czech Ministry of Health (NV19-01-00263), the 10.13039/501100001824Czech Science Foundation (22-22398S), the project National Institute for Research of Metabolic and Cardiovascular Diseases (Programme EXCELES, ID Project No. LX22NPO5104) - Funded by the European Union – Next Generation EU, and by COOPERATIO and 260646/SVV/2023 of 10.13039/100007397Charles University.

## CRediT authorship contribution statement

**Lenka Rossmeislová:** Writing – review & editing, Writing – original draft, Visualization, Validation, Supervision, Funding acquisition, Formal analysis, Data curation, Conceptualization. **Eva Krauzová:** Investigation. **Michal Koc:** Investigation, Data curation. **Marek Wilhelm:** Visualization, Investigation, Formal analysis. **Viktor Šebo:** Investigation, Formal analysis. **Zuzana Varaliová:** Investigation. **Veronika Šrámková:** Investigation. **Moniek Schouten:** Writing – original draft. **Petr Šedivý:** Investigation. **Petr Tůma:** Investigation, Funding acquisition. **Jan Kovář:** Investigation. **Dominique Langin:** Writing – review & editing, Conceptualization. **Jan Gojda:** Writing – review & editing, Supervision, Resources, Investigation, Funding acquisition. **Michaela Šiklová:** Writing – review & editing, Validation, Supervision, Funding acquisition, Data curation, Conceptualization.

## Declaration of generative AI and AI-assisted technologies in the writing process

During the preparation of this work, LR used DeepL to edit English language and ChatGPT4 in order to shorten the text and modify the Title. After using these tools, the authors reviewed and edited the content as needed and take full responsibility for the publication's contents.

## Declaration of competing interest

The authors declare that they have no known competing financial interests or personal relationships that could have appeared to influence the work reported in this paper.
